# The Changing Landscape of Invasive Fungal Infections in ICUs: A Need for Risk Stratification to Better Target Antifungal Drugs and the Threat of Resistance

**DOI:** 10.3390/jof8090946

**Published:** 2022-09-09

**Authors:** Julien Poissy, Anahita Rouzé, Marjorie Cornu, Saad Nseir, Boualem Sendid

**Affiliations:** 1 UMR 8576–UGSF–Unité de Glycobiologie Structurale et Fonctionnelle, CNRS, Pôle de Médecine Intensive-Réanimation, Inserm U1285, Université de Lille, CHU Lille, F-59000 Lille, France; 2 UMR 8576–UGSF–Unité de Glycobiologie Structurale et Fonctionnelle, CNRS, Institut de Microbiologie-Centre de Biologie Pathologie Génétique, Service de Mycologie Médicale, Inserm U1285, Université de Lille, CHU Lille, F-59000 Lille, France

**Keywords:** invasive candidiasis, candidemia, invasive aspergillosis, risk factors, antifungal, diagnosis algorithm

## Abstract

The landscape of invasive candidiasis and invasive aspergillosis has changed dramatically in intensive care units over the past two decades. Today, we are faced with new risk factors such as the emergence of resistance, but are also equipped with new therapeutic strategies and diagnostic tools which are changing epidemiological data and diagnostic algorithms. Some common points need to be addressed: (i) the best way to use microbiological tools and to integrate their results in decisional algorithms; (ii) the need to find the optimum balance between under-diagnosis and overtreatment; (iii) and the need to decipher pathophysiology. In this short review, we will try to illustrate these points.

## 1. Invasive Candidiasis

The past 20 years have been marked by a significant number of changes in the epidemiology of invasive fungal infections (IFIs) in intensive care units (ICUs), leading to many challenges.

For invasive candidiasis (IC), there has been a shift across the world to more resistant *Candida* spp., highlighting the need for well-defined strategies in order to initiate early targeted and effective antifungal therapies. Biological diagnostic tools are part of these strategies and also contribute to deciphering changes in epidemiological data.

Regarding invasive aspergillosis (IA), successive influenza and COVID-19 viral pandemics have revealed new risk profiles exposing patients to infection, in addition to standard neutropenia. This underlines the need for new diagnostic algorithms, but at the same time, raises the question of the clinical significance of the isolation of *Aspergillus* spp. from the respiratory tract.

In this review, we will discuss these issues, focusing on these two infections to illustrate the main challenges the medical community is facing today.

In this section, we will describe and analyze: (i) data regarding the incidence of IC in relation to biological tools; (ii) the most recent data regarding therapeutic strategies; (iii) the concept of risk stratification; and (iv) the most recent data regarding antifungal resistance in *Candida* spp.

### 1.1. The Challenges of Determining the True Incidence of Invasive Candidiasis and of Making a Microbiological Diagnosis

**Figure 1:**
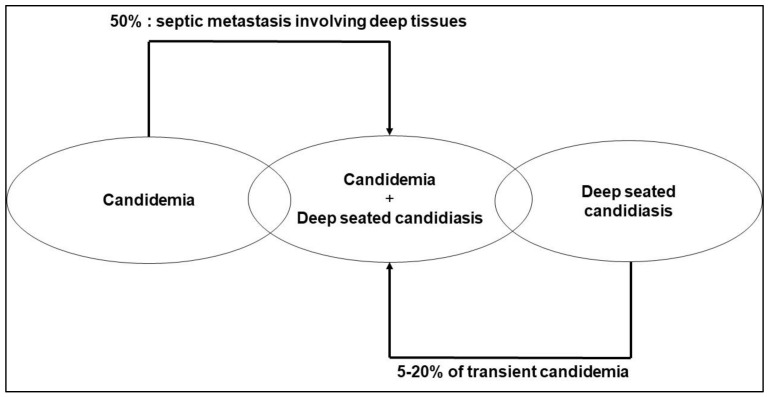
The spectrum of invasive candidiasis.

The term IC includes candidemia and deep-seated candidiasis with or without candidemia (Figure 1). *Candida* spp. are the third leading cause of nosocomial bloodstream infections but rank first in terms of mortality [1]. The incidence of candidemia has increased by 50% over the past 10 years [2,3]; this is traditionally considered to be linked to the extensive use of invasive procedures and immunosuppressive therapy. The incidence of candidemia varies between 3.5 and 16.5/1000 admissions, depending on different studies and countries [4,5,6]. A recent study showed a higher incidence of candidemia in ICUs (5.1/1000 admissions), compared to tertiary care centers (0.96/1000 admissions) [6]. Intra-abdominal candidiasis (IAC) has a lower incidence compared to candidemia alone (1.52/1000 vs. 1.84/1000 ICU admissions) [4,5]. IC has a lower incidence than bacterial infections. In a one-day prevalence, multicentric, worldwide EPIC-II study in ICUs, 50% of ICU patients were considered to be infected (7.087/13.796). *Candida* spp. were identified in 17% of microorganism-positive cultures (843/4947) isolated from these patients, ranking fourth compared to 46.8% for Gram-positive bacteria (2315/4947) and 62.2% for Gram-negative bacteria (3077/4947) [7]. However, mortality appears to be greater for *Candida* infections compared to bacterial infections. Indeed, a post-hoc study of EPIC-II showed that ICU mortality in patients with candidemia (42.6%; 26/61) was higher than mortality from bacteriemia (25.3% for Gram-positive bloodstream infections (101/420), 29.1% for Gram-negative bloodstream infections (75/264)) [5]. This high mortality rate is in accordance with other publications [8,9]. In patients with IAC, candidemia is an independent risk factor for mortality [10], suggesting that it is clinically relevant to distinguish two entities, namely IAC limited to the peritoneal cavity (and by extension deep-seated candidiasis) and IAC together with candidemia. The main limitation of studies evaluating epidemiology and risk factors is that they focus mainly on candidemia and may miss up to 50% of cases of IC due to negative blood cultures [11], as reported in old autopsy studies [12]. In IC, the sensitivity of blood cultures increases with the number of organs exhibiting lesions of deep-seated candidiasis. This lack of sensitivity is considered to be due to transient fungemia in the case of deep-seated candidiasis, or to a low fungal load and culture difficulties. However, this has been questioned due to the fact that *Candida* cell densities are low in blood samples when blood cultures are positive: 50% of first positive blood cultures from 152 episodes of candidemia had a fungal density of ≤1 colony-forming unit (CFU)/mL, challenging the molecular diagnostic tools [13,14]. One of the most recent and promising tools is the T2 Magnetic Resonance biosystem (T2MR) *Candida* panel. This combines the lysis of free DNA, lysis of the *Candida* cell wall, and molecular amplification of DNA molecules, in 5 mL of whole blood sampled in an EDTA tube, with detection based on a modification of the sample’s T2MR signal, induced by target probe-bearing nano particles coated with amplified DNA. The first lysis step explains why this tool detects DNA extracted from *Candida* cells and not free DNA [15]. All of these steps are automatized and T2MR enables the detection of the five most common species of *Candida* (group *albicans/tropicalis*, *parapsilosis*, group *glabrata/krusei*) with a range of detection of between 1 and 3 CFU/mL. T2MR cannot differentiate between *C. albicans* and *C. tropicalis*, or between *C. glabrata* and *C. krusei*. The global specificity and sensitivity per sample is >99% and >91% respectively, considering candidemia and blood cultures as the gold standard, with a mean time to negative or positive result of <5 h [16]. This raises the question of the possibility of readdressing the epidemiology of IC using new diagnostic tools. However, data about the performance of T2MR in IC are scarce. Sensitivity seems to be low, between 40 and 60% depending on the probability of the diagnosis [17]. A retrospective study based on frozen samples reported a sensitivity of T2MR of 33% in patients with IAC [18]. These data underline the huge difference between the global sensitivity evaluated on positive clinical samples and the sensitivity observed in real-life conditions. The most important benefit of this technique is the shorter time to obtain the results, which are reliable in the case of candidemia without deep-seated candidiasis. However, the gain in terms of sensitivity is still a matter of debate. It is suggested that this technique could be used in association with biomarkers (β-D-glucans), to increase the performance of these two tools in order to mutually compensate for the limitations of each test [18]. The remaining questions that need to be answered are the clinical meaning of a positive T2MR and negative companion blood cultures, probably influenced by prior antifungal therapy and neutropenia [19], and the significance of this dissociation in the follow-up of patients receiving antifungal therapy [20].

Finally, the important questions for ICU clinicians are who to treat and in whom is it possible to avoid unnecessary antifungal therapy without risk?

### 1.2. What Is New in Terms of Curative Antifungal Strategies?

The challenge is to find the optimum balance between a lack of chance and worst outcome by delaying antifungal treatment in a patient with undiagnosed IC and unnecessary treatment in a patient without IC, leading to possible side-effects, selection pressure on non-*albicans* spp., the emergence of resistance in *C. albicans* strains, and high costs. The impact of delaying antifungal treatment is well-known in candidemia: mortality of patients with candidemia varies from 10% if antifungals are introduced in the 12 h following the first positive blood culture sample versus >30% if treatment is delayed for more than 48 h [21]; in the case of septic shock, it varies from 60% at D28 if treatment is introduced in the 24 h following the diagnosis of candidemia with adequate control of the infection source, to 90% in the case of a delay of more than 24 h [22]. This probably explains why, in a one-day international study performed in ICUs, >7.5% of patients were treated with antifungals, even though there was no positive evidence for IC in two-thirds of them [23]. The results of conventional microbiological culture-based technics take too long to be compatible with early treatment, especially in the case of septic shock [24,25], and we can hypothesize that this contributes to the excessive prescription of antifungal drugs in clinical practice.

In this context, different therapeutic strategies have been developed: (i) preemptive treatment, triggered by non-culture based evidence (colonization, fungal biomarkers); (ii) empiric treatment, triggered by clinical signs of fungal infection, including fever; (iii) probabilistic treatment, based on scores or clinical rules; and (iv) definitive or targeted treatment, based on isolation of fungal agents from sterile body sites (blood culture, tissue biopsies) [26,27].

Empiric treatment was not shown to be effective in a previous study comparing fluconazole (experimental group) with placebo in ICU patients hospitalized for at least 96 h and receiving broad-spectrum antibiotherapy, with a central venous catheter and at least 4 days of fever [28]. The main reason for failure to find a difference in favor of the experimental group was the absence of resolution of fever in 55% (67/122) and 57% (73/122) of patients in the fluconazole and placebo arms, respectively. The more recent Empiricus randomized, controlled trial compared the efficacy of empiric micafungin versus placebo on fungal infection-free survival at D28 in ICU patients colonized with *Candida* spp., with acquired sepsis, multiorgan failure, on mechanical ventilation and antibiotherapy, and with central venous access. This trial did not find any benefit on survival at D28 (70% in both groups; 90/128 and 83/123, respectively), despite a significantly lower rate of at least one new fungal infection during the 28-day follow-up (IC in the micafungin arm compared to the placebo arm (3%, 4/128 vs. 12%, 15/123) [29].

The preemptive approach has also been evaluated in clinical trials. This strategy is based on the colonization index and/or polysaccharide cell wall components used as biomarkers, mainly mannan antigen/anti-mannan antibodies and β-D-glucans. Many studies in ICU patients have evaluated the sensitivity, specificity, best cut-off values for optimum sensitivity/specificity ratio, the value of marker kinetics, and the best way to combine them, based on the fact that mannan is specific while β-D-glucan is sensitive at low concentrations and specific at higher concentrations [30,31,32,33,34,35]. The negative predictive value of these biomarkers described in several studies justified using them to stop empiric treatment early in the S-TAFE randomized, controlled trial. This trial demonstrated the feasibility and efficacy of this approach by increasing the percentage of early discontinuations of antifungal therapy (54% of early discontinuations (29/54) in the biomarker driven strategy arm vs. 2% (1/55) in the routine care arm), and reducing the length of antifungal therapy (6 vs. 13 days) without any negative impact on mortality (18% and 16%, respectively), or the occurrence of subsequent IC after randomization (4% vs. 2% proven IC) [36]. Another randomized, controlled, open-label trial found similar results [37]. In accordance with these results and many other descriptive studies, the good negative predictive value of β-D-glucan enables it to rule out IC [38,39], especially in the case of low pretest probability (<5%) [40]. A higher pretest probability should trigger an alert and refers to the risk stratification discussed below. However, an alternative approach would be to determine whether it is possible to introduce preemptive antifungal treatment, based on these biomarkers. Mannan antigen lacks of sensitivity, has transient serum circulation, and is often positive late in candidemia [41]. In the Candisep trial, β-D-glucans were used as a biomarker to introduce antifungal treatment in ICU patients with sepsis and a risk of IC (experimental group) compared to a control group in which targeted antifungal therapy was driven by culture results. The median delay in introduction of antifungal treatment was significantly lower in the experimental group (1.1 day) compared to the control group (4.4 days), but without any impact on mortality at D28, which was the primary endpoint (33.7%, 58/172 in the β-D-glucan group vs. 30.5%, 51/167 in the control group). The cut-off for β-D-glucans was 80 pg/mL, but this resulted in low specificity even though the study required two successive positive results for the diagnosis of both candidemia (63.7%) and IC (65.2%), leading to antifungal use and costs that were both two-fold higher in the experimental group compared to the control group. However, the sensitivity was also very low (54.3%). Patients were mainly about to undergo surgery and were therefore not representative of a typically medical ICU cohort. The rate of IC was lower than expected and could explain this unexpected poor performance. Indeed, it has been shown that the performance of biomarkers depends on the pretest probability of IC [39,42], like other biomarkers in medicine.

In this context, it can be hypothesized that the probabilistic approach could be useful to rule out the diagnosis of IC and to stop or prevent the introduction of empiric antifungal treatment. Indeed, all clinical scores or clinical prediction rules have exhibited negative predictive values of >90%, except for peritoneal candidiasis, but low positive predictive values [43,44,45,46]. However, they have never been tested in clinical trials.

Finally, it seems the main pitfall is that the variables used to define the risk of IC in these scores or in clinical trials (sepsis, broad-spectrum antibiotics, central venous/arterial catheter, colonization, total parenteral nutrition, abdominal surgery, etc.) do not seem to be sufficiently discriminating to establish a selective risk profile. This leads to the question of risk stratification.

### 1.3. A Need for Risk Stratification to Target the Narrowest Population at High Risk

Many risk factors have been described to explain the transition from commensal gut colonization to invasive infection.

The first step in the pathophysiology of IC is the increase in colonization density, in part triggered by broad-spectrum antibiotic use. The other risk factors can be classified as: (i) a breach in the barrier defenses; and (ii) host factors increasing the risk of infection. A breach of the barrier defenses involves the skin, mucosa, and gut barriers (burns, mucositis, gastrointestinal tract perforation, intravenous lines). Host factors include comorbidities (cancer, transplant, liver and renal failure), chemotherapy/immunosuppressive drugs, and genetic susceptibility, which all lead to a defect in the immunological response [45]. The increased frequency of invasive procedures, changes in antibiotic stewardship, prevention and management of bloodstream infections secondary to intravenous lines, and use of immunomodulatory agents can lead to a change in the risk factors. A recent case-control study in this field has shown that the weight of the different clinical risk factors for candidemia differs between ICU and non-ICU patients. Cases and controls were matched for age, hospital ward, and type of surgery. Deep intravenous catheters were a risk factor in non-ICU patients but not in ICU patients. This difference could be linked to the different frequency of this variable, leading to its loss of impact in an ICU population in which it is very often used [33]. Parenteral nutrition remained a pertinent risk factor whatever the considered population. The weight of antibiotic classes was also influenced by the type of hospital ward, with glycopeptides and nitroimidazole being independent risk factors only in non-ICU patients. Finally, the concept of “broad-spectrum” was not sufficient. It can be hypothesized that this difference is due to the differences in antibiotic management according to patient profile and hospital ward, with important changes in antibiotic stewardship over the past decade in terms of spectrum and duration. Organ failure only applies to the ICU population. The global concept that emerges from these data is that we need to stratify the risk to be able to target patients with the highest prevalence and thus with the highest pretest probability. In this population, the performance of early antifungal strategies could therefore be better, especially when using the preemptive strategy [39,42]. The stratification of risk using a score with several thresholds has been evaluated in an ICU population, with the identification of three populations with three levels of prevalence and risk [47]: (i) low risk, with a prevalence of 0.24%; (ii) intermediate risk, with a prevalence of 1.46%; (iii) and high risk, with a prevalence of 11.7%. Clinicians should consider scores and biomarkers not as dichotomizing tools but as bricks to elaborate different risk strata, using the variation in performance and significance in front of the cut-off levels, as evaluated by ROC curves.

Finally, we probably need to consider two populations in ICUs, in which specific risk factor analysis will apply: (i) immunosuppressed/neutropenic patients (for whom antifungal prophylaxis is often used); and (ii) non-neutropenic and non-transplanted patients (i.e., “immunocompetent”). This latter category can be dichotomized based on the risk of deep-seated candidiasis (surgical population with the specific risk of intra-abdominal candidiasis) versus candidemia without deep-seated candidiasis. The algorithm differs between these two populations, with different performances of biomarkers allowing early withdrawal of empiric antifungal therapy on the one hand versus the introduction of preemptive therapy on the other, in a population with a high likelihood of IC after risk stratification [48]. This pragmatic approach proposed by ESICM/ESCMID experts summarizing the actual paradigm requires validation. The integration of new microbiological tools in these algorithms, such as T2MR, has to be defined.

It should be remembered that we urgently need to rationalize the use of antifungals in ICUs because of the threat of the emergence of resistance, as will be discussed in the following section.

### 1.4. Antifungal Resistance in Candida spp.

Many studies have shown that exposure to antifungal drugs plays a role in the selection of resistance, both at an individual [49] and at collective levels [50], and for both echinocandins and azoles, by promoting a switch in *Candida* spp. responsible for infection.

Over the past decade, a change in favor of non-*albicans* spp. has been confirmed in many studies worldwide, with regional heterogeneity. In Europe, *C. albicans* is still the main species isolated, in >50% of cases, while *C. glabrata* represents 15–25% of isolates, except in Spain where *C. parapsilosis* is the second most common spp. after *C. albicans*. In the USA, *C. albicans* accounts for <50% of cases and *C. tropicalis* has become to be the predominant species in Asia, especially in India and Pakistan [2]. In Latin America, the three most frequent species are *C. albicans* (around 40–45%), *C. parapsilosis* (around 25–30%), and *C. tropicalis* (around 15%), with a stable distribution in successive studies [51,52,53]. Until now, fluconazole resistance in *C. albicans* and *C. parapsilosis* has been relatively rare (4–6% worldwide), however for *C. tropicalis* it has increased to 20% in some areas, while around 10% of *C. glabrata* isolates are resistant to fluconazole [2]. A slight increase in fluconazole resistance has been described in Latin America, but it remains below 3%. Resistance to echinocandins is very rare. An increase in *C. glabrata* isolation has also been described, in Argentina, Brazil, and Colombia [54]. The variation in epidemiology worldwide can change local recommendations regarding antifungal drug use. The mechanisms of resistance vary widely, involving mainly the biosynthesis of ergosterol by mutations in the ERG11 gene, leading to drug target overexpression or alterations, but also involving bypass pathways, efflux pump overexpression, and aneuploidy/loss of heterozygosity [55]. Guidelines have endorsed the use of echinocandins as first-line therapy because of this evolving rate of resistance, fungicidal versus fungistatic activity, and less drug-drug interactions [26,48,56,57]. However, de-escalation to fluconazole must be discussed when susceptibility is known in clinically stable patients. Indeed, the extensive use of echinocandins increases the risk of echinocandin resistance, as described in 9% of fluconazole resistant *C. glabrata* strains, even though resistance to echinocandins in *C. glabrata* and *C. albicans* strains is currently rare [2,58]. In this context, the follow-up of trends in resistance is very important, and new tools such as MALDI-TOF could help to obtain quick results, which will enable the adaptation of therapeutic schedules at an individual level as quickly as possible [59], while taking into account that pre-exposure to azoles has significantly increased over the past 20 years [60].

The most frightening threat in the field of antifungal resistance is the emergence of *C. auris* [61,62]. This species was first identified in 2009 and has since been involved in many outbreaks worldwide. This species colonizes the skin but not the gut. It can remain viable for long periods on environmental surfaces, causing local dissemination. Strains are mostly susceptible to echinocandins, but resistance is frequent to azoles and amphotericin B. Up to 40% of isolates are resistant to at least two antifungals [63], and pan-resistance has also been described in some clinical strains [64]. However, the majority of patients are colonized, but not infected by this strain. In a cohort of ICU patients, 17% of patients colonized with *C. auris* subsequently developed candidemia. For more than 25% of these patients, candidemia occurred >60 days after the first isolation [65]. This underlines the fact that infections seem to occur late in the history of hospitalization, in frail patients. The challenges are then to identify outbreaks and to control them by a stepwise and multidisciplinary process, to distinguish colonization from infection at the individual level [66], and to decrease antifungal pressure by rationalizing their prescription, as stated above.

## 2. Invasive Aspergillosis: Determining the Reality of Infection in New Risk Profiles

In this section, we will describe and analyze: (i) what has changed in the immuno-suppressed patient paradigm; (ii) how influenza has modified the diagnosis of IA; and (iii) the questions posed by IA in patients with COVID-19.

### 2.1. Changes in the Immunosuppressed Patient Paradigm

During the last two decades, the epidemiological landscape of IA has changed dramatically in ICUs, resulting in important changes in diagnostic algorithms. Twenty years ago, this disease was mainly seen in immunosuppressed patients, such as those suffering from hematological disorders and cancer, especially during periods of neutropenia. In this category of patients, diagnosis is performed using EORTC rules, in which entry is determined by immunosuppression, the definition of which has been regularly updated to take into account new drugs and treatments, as well as new microbiological tools. Radiological phenotypes, like clinical factors, also play an important role in this specific algorithm, along with host and microbiological factors [67], to classify patients into those with proven, probable, or possible IA. The prevalence of IA has decreased as a result of antifungal prophylaxis, but this disease is still associated with high mortality rates [68]. In a post-mortem series of patients with a proven diagnosis, it has been shown that the phenotypes of infected patients with hematological disorders have changed with a progressive switch over the past two decades from neutropenic patients to patients receiving high doses of corticosteroids, or with graft versus host disease [69]. These different sub-clinical backgrounds and phenotypes are supported by different physiopathological patterns in experimental models. Although neutropenia is associated with the absence of recruitment of immune cells and a lack of inflammatory response, bleeding, proliferation and dissemination of mycelial forms of the fungus, corticosteroids are associated with neutrophil infiltration, alveolar bleeding, exacerbation of the inflammatory response, and low proliferation of the fungus with few hyphal forms [70]. These data support the diverse patterns of the disease.

In addition to this change in phenotype of hematological patients, new categories of non-hematological patients suffering from IA have also been described in ICUs, including COPD, cirrhosis, and acute hepatitis [71,72,73,74,75]. These patient profiles have supported the idea that the continuum between abnormal colonization of the respiratory tract to invasive/angio-invasive fungal disease can occur in patients with alterations in mucociliary clearance and/or with different alterations in immune response outside neutropenia. The inflammatory spectrum of lung diseases due to *Aspergillosis* spp., such as allergic bronchopulmonary aspergillosis [76], is not discussed in this review.

The identification of new categories of patients suffering from IA has led to a new definition, putative aspergillosis, and a new diagnostic algorithm for ICU patients, Asp-ICU [77]. The most important changes in the way in which a positive diagnosis is considered are: (i) the entry criterion is an *Aspergillus*-positive endotracheal aspirate culture and not a host criterion. This means that ICU clinicians should keep an open mind when diagnosing IA in a patient with positive cultures without the classic criteria of immunosuppression. However, the risk is to overtreat patients considered to have IA whereas they are only colonized; (ii) to avoid this last pitfall, the second criterion also considers clinical and radiological signs of infections. This can be problematic in patients with preexisting lung diseases (such as infections); (iii) host criteria can be replaced by semi-quantitative cultures showing a high fungal load. In other words, this algorithm introduces the concept of fungal density in the lung by culture-based microbiological techniques, to differentiate between colonization and infection.

The Asp-ICU algorithm was tested in patients with histopathological evidence of IA and was significantly more accurate than EORTC criteria in a non-neutropenic ICU population (area under the ROC curve (AUC) for Asp-ICU = 0.76; AUC for EORTC criteria = 0.57).

In order to improve the accuracy of this algorithm, tests for galactomannan (GM), a cell wall polysaccharidic component specific to *Aspergillosis* spp. were carried out in ICU patients at risk of IA. The cohort evaluated consisted of 25% hematological patients and 75% without cancer or hematological disorders. The detection of GM in bronchoalveolar lavage fluid (BALF) had a sensitivity of 88% and a specificity of 87% for the diagnosis of IA in 110 patients considered at risk, among which 26 cases of IA were proven. GM detection was not accurate when tests were carried out on serum. Interestingly, almost 50% of proven IA cases had negative BALF cultures, whereas GM was positive in BALF (11/26 proven cases) [78]. GM in BALF probably reflects the hyphal proliferation of *Aspergillus* spp., correlating with the pathological invasion of lung tissue, and appears to be an interesting theoretical tool to differentiate colonization from infection. From the pathophysiological point of view, a positive GM test in serum probably indicates the angio-invasive form of the disease and has a specific value despite its lack of sensitivity.

### 2.2. The Revolution of the Influenza Algorithm

GM was included in the criteria to evaluate the possibility of IA in ICU patients hospitalized with severe influenza. Using this tool, influenza has been described as an independent risk factor for IA, which affected nearly 20% of patients [79]. In this retrospective, cohort study, influenza increased the risk of IA almost three-fold compared to non-immunosuppressed patients without influenza. Influenza-associated pulmonary aspergillosis (IAPA) occurred with a median delay of 3 days after ICU admission, and the impact on mortality was very high (47% in patients without underlying immunosuppression, 71% in immunosuppressed patients). GM was positive in 92% of BALF samples and in 65% of serum specimens, contrasting with previous findings. This is an argument for an angio-invasive process by epithelial destruction. Influenza was also associated with a proximal form of IA, tracheobronchitis, diagnosed by fibroscopy, with a suggestive macroscopic appearance and microbiological confirmation [79]. This entity has allowed us to understand the role played by this virus, which destroys the respiratory epithelium and facilitates tissue invasion by *Aspergillus*. Moreover, patients with severe influenza can present with post-aggressive immune-paralysis [80], underlining the fact that “immunosuppression” is not limited to immunosuppressive drugs, cancer, or transplantation.

By contrast, a retrospective French study, using the Asp-ICU algorithm without a GM test, found a prevalence of IAPA of only 1.6% (10/524 patients hospitalized with severe influenza, among which 28 patients had positive respiratory tract samples for *Aspergillosis* spp.) [81], questioning the best criterion and the role of GM in making the diagnosis. The pragmatic specific algorithm proposed by a group of multidisciplinary experts introduces the use of GM as the first biological criterion to enter into the algorithm. Radiological signs, which are difficult to analyze in the context of severe influenza, are used only in cases with positive *Aspergillus* cultures from sputum or endotracheal aspirates (and not BALF) with negative GM [82]. This algorithm, using GM, still needs to be validated using the histopathological gold standard.

Interestingly, the relatively high prevalence of IAPA has not been significantly decreased by antifungal prophylaxis with posaconazole, tested in a pilot, clinical trial [83]. IAPA was diagnosed in the first 48 h after admission to an ICU in 71% of patients (15/21), out of a total of 88 patients included. This prevalence of 24% was higher than anticipated and the patients already infected in the first 48 h could not be analyzed. These data suggest that we currently lack the tools to identify patients at high-risk of IAPA, in order to treat them early.

### 2.3. CAPA: An Illustration of Cognitive Bias?

Probably because of reasoning by analogy, the COVID-19 pandemic has generated a plethora of publications on IA associated with this new respiratory viral infection, defining COVID-19 associated pulmonary aspergillosis (CAPA). Data are contradictory, showing a prevalence ranging from 4.8% to 23% in ICU patients. This is probably due to the great variability in definitions and diagnostic criteria used, some confusion in the denominator [84,85], confusing variables in underlying conditions [86], misevaluation due to temporal variations in the diagnostic tools used at the bedside (BALF), and environmental contamination (extensive use of negative pressure rooms) [71]. In a cohort of 509 patients receiving invasive mechanical ventilation for more than 48 h, in whom at least three samples were analyzed for *Aspergillus*, 14.9% developed proven or probable CAPA [87]. The incidence of CAPA is probably overestimated in this study, due to the denominator of patients with at least three samples even though the population at risk included all COVID-19 patients receiving invasive mechanical ventilation. The most stringent studies used the Blot Asp-ICU algorithm [88], or EORTC criteria for immunosuppressed patients and putative/probable categories for non-immunosuppressed subjects, with the exclusion of patients with a single positive non-culture-based fungal diagnostic test or an isolated positive fungal culture with negative follow-up cultures [86]. They found a prevalence of CAPA lower than that of IAPA (<5%). The disease was rare in non-immunosuppressed patients. The prevalence of CAPA should be reevaluated with the extensive use of immunomodulatory drugs such as corticosteroids or various anti-interleukin drugs.

A recent meta-analysis including eight cohort studies and 729 critically ill COVID-19 patients reported that CAPA patients were older, had underlying COPD, and were more likely to receive long-term corticosteroid treatment compared to COVID-19 patients without CAPA. CAPA patients had also a greater Sequential Organ Failure Assessment score with a higher all-cause in-hospital mortality rate (42.6% vs. 26.5%; Odds ratio = 3.39; *p* < 0.001) [89]. As on other wards treating at risk patients, ICU-related factors such as environmental factors, isolation conditions, ventilation systems, building renovation works, and temporal spread with respect to pandemic waves, should be considered when evaluating the risk of CAPA [90]. The delay in occurrence of CAPA appeared to be longer than that reported for IAPA.

An observational study of 135 COVID-19 patients found a significantly lower incidence of CAPA in patients who received prophylactic posaconazole compared to those who did not [91]. However, this did not translate into improved survival. Another retrospective study suggested a beneficial effect of aerosolized liposomal amphotericin-B in preventing CAPA and *Aspergillus* tracheobronchitis [92]. Randomized, controlled trials are required to evaluate the efficacy and safety of prophylactic antifungal treatment in COVID-19 patients.

In all of these studies, few pathological data are available to determine whether CAPA is a new entity or not. Despite this lack of evidence, several expert recommendations have been made [93,94], integrating new biological tools such as PCR but also non- validated cut-offs to distinguish colonization from infection. These approaches are promising and appealing, however only the Asp-ICU algorithm has been validated by histopathological confrontation. This raises the question of the timing of implementation of biological tools in guidelines before their strict clinical validation [95].

Considering colonization as the first step in the pathological process, the question of the timing of treatment initiation remains. The preemptive strategy with early treatment withdrawal could be an option, as suggested by several authors [93,96], but needs to be evaluated. Like IC, this type of approach would benefit from risk stratification and the validation of algorithms based on pretest probability.

## 3. Conclusions: Different Invasive Fungal Invasions, the Same Pitfalls and Challenges

The examples described here for IC and IA shed light on a number of common problems (Table 1), namely: (i) The need for risk stratification to target groups of patients with the highest risk of IFI; (ii) The need to describe and better understand the pathophysiology of IFI. Histopathological confirmation still plays an important role in this field; (iii) The need to assess the performance and significance of new biological tools before introducing them in new guidelines; (iv) The misuse of new sensitive tools should be avoided, because it could lead to the overestimation of the real prevalence of a disease and overtreatment, which would favor the emergence of resistance.

All of these considerations apply to other fungal diseases [71]. In the past, many diagnoses of IFI have been missed and patients have been undertreated. In contrast, we are now faced with the overestimation of IFIs and the overuse of antifungal drugs.

This highlights the importance of a multidisciplinary approach to the management of IFIs, taking into account clinical, radiological, histological, and biological data.

## Figures and Tables

**Table 1:** Common pitfalls and challenges of invasive candidiasis and invasive aspergillosis in ICU patients.

	Invasive Candidiasis			Invasive Aspergillosis		
	*Old Concepts–*	*New Concepts–*	*Future Challenges–*	*Old Concepts–*	*New Concepts–*	*Future Challenges–*
Epidemiology/ diagnosis	- Gold standard = histopathology - Bedside gold standard = blood cultures - 50% of IC misdiagnosed by blood cultures	- New microbiological tools could help to improve the diagnosis of IC: biomarkers/T2MR	- To evaluate and integrate these tools and update the epidemiology	- Gold standard = histopathology - Bedside gold standard = BALF cultures - Classification proven/probable/possible - How to differentiate colonization from infection?	- Putative IA - New microbiological tools could help to improve the diagnosis of IA: biomarkers, molecular biology	- To evaluate and integrate these tools to update the epidemiology - Histopathological confrontation needed
Risk factors	- Colonization - Breach of barrier defenses - Host factors	- Relevance of risk factors depends on the sub- population	- Stratify the group with the highest prevalence and the highest pretest probability of IC	Immunosuppression	- Alteration of mucociliary clearance - Post- aggressive immune- paralysis - Viral aggression?	Stratify the risk
Treatment	- Treating proven IC is too late - Empiric strategy	- Empiric strategy is not efficient - Early withdrawal of empiric strategy is possible	- Rationalization of antifungal use - Define better strategies to introduce antifungals Survey and control resistance emergence	Treating proven IA is not sufficient		- Balance between under-diagnosis and overtreatment - Define empiric/ preemptive/definitive treatments?

IA: invasive aspergillosis; IC: invasive candidiasis; ICU: intensive care unit; BALF: bronchoalveolar lavage fluid.

## Data Availability

Not applicable.
